# Supplementary figures and images for: Prognostic Impact of Pulmonary Metastasectomy in Bone Sarcoma Patients: A Retrospective, Single-Centre Study
Source: Cancers (Basel). 2023 Mar 13;15(6):1733. doi: 10.3390/cancers15061733 (PMC10046382; doi:10.3390/cancers15061733)

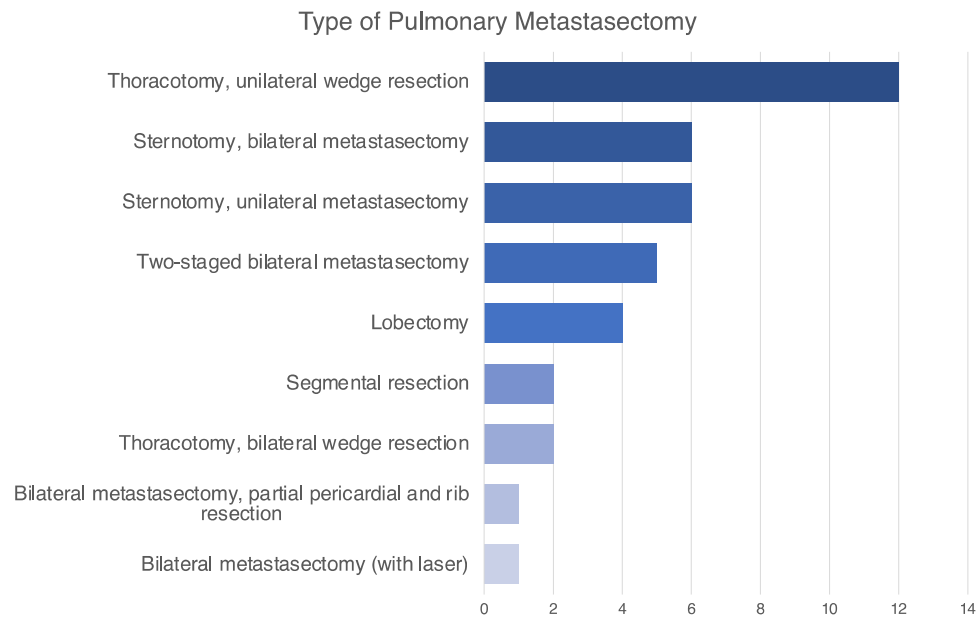

**Figure S1.** Type of pulmonary metastasectomy.

Supplement: Supplementary file 1 [file cancers-15-01733-s001.zip › cancers-2261950-supplementary.pdf]
